# Supplementary material for: COZOID: contact zone identifier for visual analysis of protein-protein interactions
Source: BMC Bioinformatics. 2018 Apr 6;19:125. doi: 10.1186/s12859-018-2113-6 (PMC5889581; doi:10.1186/s12859-018-2113-6)
Supplement: Supplementary file 2 — Software build. Executable binary file of the software tool. (ZIP 118,784 kb) [file 12859_2018_2113_MOESM2_ESM.zip › cozoid/java/docs/beansbinding-1.2.1-doc.zip!/index-files/index-20.html]

T-Index (Beans Binding)


---


|  |  |  |
| --- | --- | --- |
| **Overview** \| Package \| Class \| Use \| **Tree** \| **Deprecated** \| **Index** \| **Help** | |  |
| **PREV LETTER**   **NEXT LETTER** | **FRAMES**    **NO FRAMES**     **All Classes** |  |


A B C D E F G H I J K L M N O P Q R S T U V W 

---


## **T**

**target** - Variable in class org.jdesktop.el.impl.lang.FunctionMapperFactory: **targetChanged(Binding, PropertyStateEvent)** - Method in class org.jdesktop.beansbinding.AbstractBindingListener: Notification that the target property of a `Binding` has fired a `PropertyStateEvent` for the `Binding's` target object. **targetChanged(Binding, PropertyStateEvent)** - Method in interface org.jdesktop.beansbinding.BindingListener: Notification that the target property of a `Binding` has fired a `PropertyStateEvent` for the `Binding's` target object. **targetChangedImpl(PropertyStateEvent)** - Method in class org.jdesktop.beansbinding.AutoBinding: **targetChangedImpl(PropertyStateEvent)** - Method in class org.jdesktop.beansbinding.Binding: Called to indicate that the target property has fired a `PropertyStateEvent` to indicate that its state has changed for the target object. **targetEdited(Binding)** - Method in class org.jdesktop.beansbinding.AbstractBindingListener: **Deprecated.** *This method has been replaced by `AbstractBindingListener.targetChanged(org.jdesktop.beansbinding.Binding, org.jdesktop.beansbinding.PropertyStateEvent)` and it will go away soon. It is being kept for a short period only, to assist in migration.* **throwIfBound()** - Method in class org.jdesktop.beansbinding.Binding: Throws an IllegalStateException if the `Binding` is bound. **throwIfManaged()** - Method in class org.jdesktop.beansbinding.Binding: Throws an UnsupportedOperationException if the `Binding` is managed. **throwIfUnbound()** - Method in class org.jdesktop.beansbinding.Binding: Throws an IllegalStateException if the `Binding` is unbound. **throwUnhandled(Object, Object)** - Static method in class org.jdesktop.el.impl.lang.ELSupport: **toFloat(String)** - Static method in class org.jdesktop.el.impl.lang.ELSupport: **token** - Variable in class org.jdesktop.el.impl.parser.ELParser: **Token** - Class in org.jdesktop.el.impl.parser: Describes the input token stream. **Token()** - Constructor for class org.jdesktop.el.impl.parser.Token: **token\_source** - Variable in class org.jdesktop.el.impl.parser.ELParser: **tokenImage** - Static variable in interface org.jdesktop.el.impl.parser.ELParserConstants: **tokenImage** - Variable in exception org.jdesktop.el.impl.parser.ParseException: This is a reference to the "tokenImage" array of the generated parser within which the parse error occurred. **TokenMgrError** - Error in org.jdesktop.el.impl.parser: **TokenMgrError()** - Constructor for error org.jdesktop.el.impl.parser.TokenMgrError: **TokenMgrError(String, int)** - Constructor for error org.jdesktop.el.impl.parser.TokenMgrError: **TokenMgrError(boolean, int, int, int, String, char, int)** - Constructor for error org.jdesktop.el.impl.parser.TokenMgrError: **toNumber(String)** - Static method in class org.jdesktop.el.impl.lang.ELSupport: **toString()** - Method in class org.jdesktop.beansbinding.BeanProperty: Returns a string representation of the `BeanProperty`. **toString()** - Method in class org.jdesktop.beansbinding.Binding.SyncFailure: Returns a string representation of the `SyncFailure`. **toString()** - Method in class org.jdesktop.beansbinding.Binding: Returns a string representation of the `Binding`. **toString()** - Method in class org.jdesktop.beansbinding.Binding.ValueResult: Returns a string representation of the `ValueResult`. **toString()** - Method in class org.jdesktop.beansbinding.ELProperty: Returns a string representation of the `ELProperty`. **toString()** - Method in class org.jdesktop.beansbinding.ObjectProperty: Returns a string representation of the `ObjectProperty`. **toString()** - Method in class org.jdesktop.beansbinding.PropertyStateEvent: Returns a string representation of the `PropertyStateEvent`. **toString()** - Method in class org.jdesktop.beansbinding.Validator.Result: Returns a string representation of the `Result`. **toString()** - Method in class org.jdesktop.el.impl.parser.AstFunction: **toString()** - Method in class org.jdesktop.el.impl.parser.SimpleNode: **toString(String)** - Method in class org.jdesktop.el.impl.parser.SimpleNode: **toString()** - Method in class org.jdesktop.el.impl.parser.Token: Returns the image. **toString()** - Method in class org.jdesktop.el.impl.ValueExpressionImpl: **toTypeArray(String[])** - Static method in class org.jdesktop.el.impl.util.ReflectionUtil: Converts an array of Class names to Class types **toTypeNameArray(Class[])** - Static method in class org.jdesktop.el.impl.util.ReflectionUtil: Converts an array of Class types to Class names **TRUE** - Static variable in interface org.jdesktop.el.impl.parser.ELParserConstants: **TYPE** - Static variable in class org.jdesktop.el.ELResolver: The attribute name of the named attribute in the `FeatureDescriptor` that specifies the runtime type of the variable or property. **types** - Variable in class org.jdesktop.el.impl.lang.FunctionMapperImpl.Function

---


|  |  |  |
| --- | --- | --- |
| **Overview** \| Package \| Class \| Use \| **Tree** \| **Deprecated** \| **Index** \| **Help** | |  |
| **PREV LETTER**   **NEXT LETTER** | **FRAMES**    **NO FRAMES**     **All Classes** |  |


A B C D E F G H I J K L M N O P Q R S T U V W 

---
